# O-GlcNAcylation: The Sweet Side of the Cancer

**DOI:** 10.3389/fonc.2014.00132

**Published:** 2014-06-03

**Authors:** Rafaela Muniz de Queiroz, Érika Carvalho, Wagner Barbosa Dias

**Affiliations:** ^1^Laboratório de Glicobiologia Estrutural e Funcional, Centro de Ciências da Saúde – Bloco G, Instituto de Biofísica Carlos Chagas Filho, Universidade Federal do Rio de Janeiro, Rio de Janeiro, Brazil; ^2^Instituto de Bioquimica Médica, Universidade Federal do Rio de Janeiro, Rio de Janeiro, Brazil

**Keywords:** *O*-GlcNAc, O-GlcNAcylation, cancer, review, tumorigenesis, metabolism, tumor biology, metastasis

## Abstract

O-GlcNAcylation is an *O*-linked β-*N*-acetylglucosamine (*O*-GlcNAc) moiety linked to the serine or threonine residues in proteins. O-GlcNAcylation is a dynamic post-translational modification involved in a wide range of biological processes and diseases such as cancer. This modification can increase and decrease the activity of enzymes as well as interfere with protein stability and interaction. The modulatory capacity of O-GlcNAcylation, as well as protein phosphorylation, is of paramount importance in the regulation of metabolism and intracellular signaling of tumor cells. Thus, understanding the regulation of O-GlcNAcylation in tumor cells and their difference compared to non-tumor cells may elucidate new mechanisms related to tumor generation and development, could provide a new marker to diagnosis and prognosis in patients with cancer and indicate a new target to cancer chemotherapy.

## Introduction

*O*-linked β-*N*-acetylglucosamine (*O*-GlcNAc) was discovered 30 years ago (1). Unlike “traditional glycosylation,” *O*-GlcNAc is not elongated into more complex structures and is localized mainly in nucleocytoplasmic compartments. The addition of *O*-GlcNAc to proteins is catalyzed by *O*-GlcNAc transferase (OGT), and the *O*-GlcNAcase (OGA) catalyzes its removal (2). Deletion of OGT is lethal in mice at the embryonic level and at the single-cell level, highlighting the importance of O-GlcNAcylation in regulating basic cellular events (3). Similar to phosphorylation, *O*-GlcNAc is an abundant, dynamic, and inducible post-translational modification (PTM) occurring on serine and threonine residues. The relationship between phosphorylation and O-GlcNAcylation is more complex than initially thought not only in terms of site occupancy, where both PTMs occur on Ser/Thr and can modulate each other (2) but also *O*-GlcNAc can regulate Tyr phosphorylation, indicating that interplay between these PTMs at the substrate level is not limited to Ser and Thr residues (4). Therefore, growing evidences suggest that O-GlcNAcylation regulate kinases and/or phosphatases (5–7), making the relationship between these two PTMs even more complex. Approximately 1000 proteins have been described to be *O*-GlcNAcylated to date (8). Like phosphorylation, O-GlcNAcylation can modulate protein function, including enzymatic activity, protein turnover, protein interactions, subcellular localization, DNA affinity, and transcription activity (2).

The hexosamine biosynthetic pathway (HBP) is a branch of the glucose metabolic pathway, consuming approximately 2–5% of the total glucose (9). The first and limiting step of the HBP is catalyzed by glutamine: fructose-6-phosphate amidotransferase (GFAT) that converts fructose-6-phosphate to glucosamine-6-phosphate with the concomitant conversion of glutamine to glutamate. Glucosamine-6-phosphate is further metabolized to UDP-GlcNAc, which serves as the monosaccharide donor for O-GlcNAcylation. UDP-GlcNAc is considered an ideal sensor for the metabolic status of the cell, as it requires glucose, glutamine, acetyl-Coenzyme-A, and nucleotide uridine-5′-triphosphate (UTP). Nutrient-sensitive changes the flux through the HBP either increasing or decreasing UDP-GlcNAc levels, affecting the O-GlcNAcylation of many proteins, since OGT is highly sensitive to UDP-GlcNAc levels across a very broad range of concentrations (10). Several *O*-GlcNAcylated proteins are involved in the development and pathology of major diseases such as cancer, diabetes and Alzheimer’s disease (11, 12).

**Figure 1:**
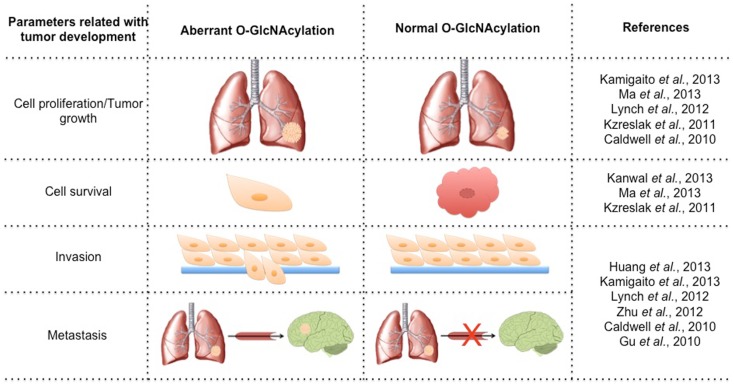
**Variation of the parameters involved in tumorigenesis by modulating the levels of O-GlcNAcylation**.

**Figure 2:**
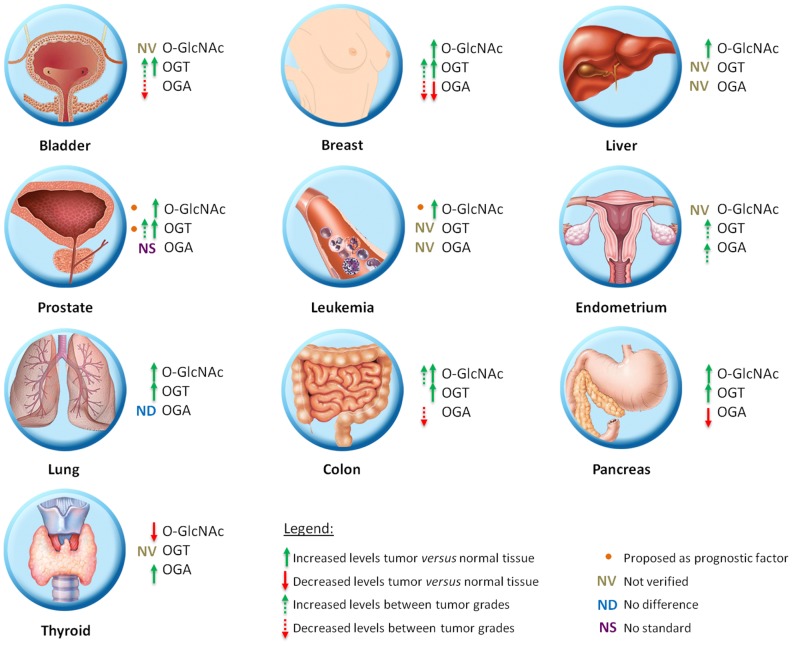
**Schematic representation of *O*-GlcNAc dynamics in different types of tumor**.

Cancer is a multifactorial disease characterized by the uncontrolled proliferation of cells. Tumor cells have altered glucose metabolism, producing ATP primarily through glycolysis, even under normoxic conditions. This metabolic shift was termed the “Warburg effect” and involves up-regulation of glucose uptake and is critical for supporting a malignant phenotype (13). The high rate of glycolytic flux is a central metabolic hallmark of tumors. An increase of about 10-fold more glucose incorporation has been demonstrated in several types of human cancers than in adjacent normal tissue. Increased expression of GLUT1 has been shown in various types of cancer (14). This phenomenon of elevated glucose uptake has been clinically exploited to detect tumor cells by positron emission tomography (PET) scans (15). Glutamine is a key nutrient for tumors cells, being a major source of nitrogen and energy in rapidly dividing cells. Recently, Itkonen and colleagues showed that several HBP genes were overexpressed in human prostate cancers (16). This phenomenon has been speculated for several years due to the suggestive increased uptake of glucose and glutamine in tumor cells. The link between altered metabolism and glycosylation through HBP provides a mechanism for the cancer cells to sense and respond to a variety of environmental conditions. It is already known that HBP flux can alters cell surface *O*-Glycans (17) and *N*-Glycans (18), but our focus here will be *O*-GlcNAc. Several studies have related parameters of tumor development with *O*-GlcNAc (Figure 1). Many signaling pathways become deregulated during the transformation due to a gain of function of oncogenes and a loss of function of tumor suppressors. O-GlcNAcylation modulates several tumor-associated proteins including p53, c-Myc, beta-catenin, Ras, and NF-κB, among others. About 60 papers have been published in the last 3 years relating *O*-GlcNAc and cancer. A substantial number of them have related the increase of *O*-GlcNAc and OGT in several types of tumors [for review see Ref. (19, 20)]. Many authors have reported that the levels of *O*-GlcNAc and the protein expression of OGT and OGA are aberrant in different models, including cell lines, murine models, and human tumor samples. Recently, some authors have associated O-GlcNAcylation with prognosis and tumor grade. Below, we summarize the changes of *O*-GlcNAc levels and the cycling enzymes in different tumor types (Figure 2).

## *O*-GlcNAc Dynamics in Different Tumor Types

### Breast cancer

Alterations in *O*-GlcNAcylated protein levels as well as OGT and OGA levels are well established for breast cancer. Studies show that both cell lines and patient samples contain elevated O-GlcNAcylation and increased OGT levels (21–23). Gu and colleagues (21) showed by immunochemistry that *O*-GlcNAc modification is elevated in human breast tumor tissue when compared with adjacent normal tissue. In the same direction, Krzeslak et al. (24) demonstrated that patient samples have more OGT mRNA and less OGA mRNA than normal samples. In addition, Champattanachai and colleagues (23) confirmed by immunoblotting that patient samples have elevated levels of OGT and *O*-GlcNAc when compared with normal patient samples. When analyzing breast tumor cell lines, Caldwell et al. (22) showed the same correlation between normal and tumor cells, where the tumor cell lines appeared to contain more *O*-GlcNAc modification and elevated levels of OGT when compared to normal cell lines. There was also an association between *O*-GlcNAc, OGT, and OGA levels with breast tumor malignancy. Tumor progressions were accompanied by increased levels of OGT mRNA and decreasing OGA mRNA (24). Champattanachai et al. (23) showed an increase of OGT protein and *O*-GlcNAc levels related to the histological grade of breast tumors. In oppose direction, Slawson and colleagues (25) showed, in a reduced sample size, an increase in OGA activity with breast cancer tumor grade.

### Prostate cancer

Lynch and colleagues (26) showed that *O*-GlcNAc and OGT levels are higher and OGA/OGT ratios are lower in tumor cells than in normal cells lines. This diminished ratio may lead to the higher O-GlcNAcylation. In addition, they examined OGT expression using the Oncomine™ Database and found elevated OGT mRNA expression patterns in human prostate carcinoma compared with normal adjacent tissue in more than 200 patient samples. With respect to tumor progression, the authors searched the National Center for Biotechnology Information (NCBI) database and found a positive correlation between high OGT expression and metastatic progression in normal, primary tumor, and metastatic prostate tumor tissues. The OGT levels were also analyzed as prognostic factors and it was observed that disease-free survival 5 years post-treatment for prostate cancer was higher in patients with a low OGT expression profile compared with patients with increased OGT expression (26). In this direction, Kamigaito and colleagues (27) also proposed that the increase of *O*-GlcNAc levels as a prognostic factor, because they observed a significant association between this PTM and a poor patient prognosis in a 5-year overall survival analysis in 56 patients.

### Colon cancer

Studies showed an increase of *O*-GlcNAc and OGT levels in patient samples by immunochemistry and immunoblotting, but OGA levels were not modified when compared with normal colon tissue (28, 29). Using primary and metastatic colon cancer cell lines derived from the same patient, Yehezkel and colleagues showed that metastatic cells have increased levels of *O*-GlcNAcylated proteins and reduced OGA, indicating a relationship with malignity (30). However, no significant difference between the cell lines was found for OGT. They also showed that both OGT and OGA are more readily detected in the cytoplasm than in the nucleus of colon tumor cells (30).

### Bladder cancer

Studies involving *O*-GlcNAc and bladder cancer are directed toward diagnosis and prognosis. Investigation of the presence of the mRNA of *O*-GlcNAc cycling enzymes was performed by real-time PCR assay using the urine of 176 patients and 143 health persons. OGT mRNA was present in more than half of the urine samples from bladder cancer patients, but was not found in the urine of healthy patients. Therefore, the analysis of urinary content of OGA and OGT mRNA may be useful for bladder cancer diagnostics. Moreover, OGT expression was significantly higher in grade II and III in comparison to grade I bladder cancer indicating a potential in prognosis (31).

### Leukemia

The *O*-GlcNAc dynamics in a leukemia model is poorly known, but chronic lymphocytic leukemia (CLL) patients present with increased *O*-GlcNAc levels in lymphocytes when compared with those from normal blood samples. OGT and OGA levels were not investigated. However, unlike the results observed in other tumor types, low *O*-GlcNAc levels were associated with more aggressive CLL (32).

### Endometrial cancer

Krzeslak and colleagues (33) determined the *O*-GlcNAc cycling enzymes mRNA levels for endometrial cancer using real-time RT-PCR analysis of 76 patient samples. Both OGT and OGA mRNA expression was significantly higher in high grade tumors than in low grade ones. They also showed an association between OGT and OGA expression and myometrial invasion (33), indicating that O-GlcNAcylation can be related to aggressiveness.

### Liver cancer

Liver transplantation represents the treatment of choice for patients with early stage of hepatocellular carcinoma (HCC). However, frequent tumor recurrence following transplant remains the major obstacle for long-term survival. Zhu et al. (34) demonstrated that patients who had suffered from tumor recurrence after liver transplantation during the follow-up period had an enhanced O-GlcNAcylation level compared to those who had been recurrence free. Patients with low OGA expression in tumors had an increasing risk of tumor recurrence after liver transplantation, indicating that OGA expression may have prognostic value for HCC. OGT expression was not correlated with HCC prognosis (34).

### Pancreatic cancer

Following the patterns seen for other tumors, analysis of *O*-GlcNAc levels in pancreatic tumors and normal patient tissues by immunochemistry showed that tumor samples have increased levels of *O*-GlcNAc. Measuring normal pancreas and tumor cell lines showed that OGT protein levels in tumors were higher than in normal cell lines and that OGA levels were decreased (35).

### Lung cancer

Immunochemistry analysis of patient samples showed that *O*-GlcNAc and OGT has the same alterations found in other tissues, with increased protein levels in tumor tissues when compared with adjacent healthy tissue, and that OGA levels appear to be unaltered (28).

### Thyroid cancer

In oppose direction, Krzeslak et al. (36) showed that *O*-GlcNAcylated protein levels are decreased in thyroid tumors and accompanied by an increase of OGA activity. They also observed that most of the *O*-GlcNAcylated proteins were found in the nucleus.

These studies demonstrate that O-GlcNAcylation is indeed altered in tumors in general when compared to normal tissues. However, there is no general rule about the variation in the levels of this modification or its relationship with prognosis, since different tumor tissues may have different levels of *O*-GlcNAc and influence on prognosis.

## *O*-GlcNAc in Metabolic Reprograming

**Figure 3:**
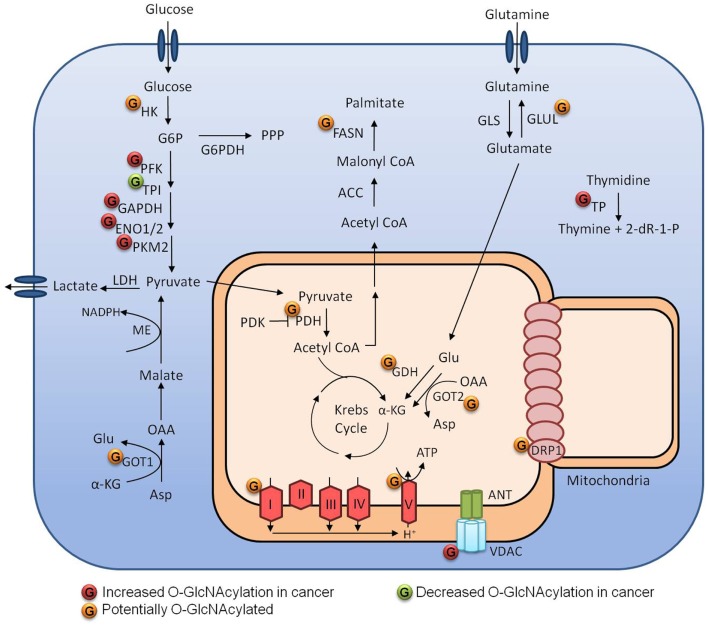
**Metabolism regulation by O-GlcNAcylation in tumor cells**.

The main carbon source of cancer cells is glucose, and besides increased glucose uptake, cancer cells display increased glycolysis and lactate production, even in the presence of oxygen; this is known as the Warburg effect. This phenotype is important as it provide intermediates for other anabolic pathways. Metabolic reprograming is an essential characteristic achieved by cancer cells to support proliferation (37). Tumor cells are often exposed to a heterogeneous microenvironment with different nutrient and oxygen availabilities (38). O-GlcNAcylation is a nutrient-sensitive PTM that responds to changes of the sugar donor UDP-GlcNAc. Growing evidence indicates that *O*-GlcNAc is a crucial mechanism linking cancer metabolism and cellular signaling (Figure 3) (16, 19, 20).

### Glycolysis

As a point of convergence of metabolic pathways, it is expected that O-GlcNAcylation can exert a strong influence on cellular metabolism and the enzymes that perform catalysis. Not surprisingly, several glycolytic enzymes can be *O*-GlcNAcylated (23, 39). Yi et al. (40) demonstrated that both OGA inhibition and OGT overexpression decreased PFK1 activity, as well as glycolytic rate and lactate production. They demonstrated that PFK1 O-GlcNAcylation at serine 529 was essential to decrease the PFK1 activity, diverting glucose-6-phosphate to the pentose phosphate pathway (PPP), and conferring a growth advantage to cancer cells by producing more NAPDH, which is an important component of the antioxidant system. In the same report, they examined the impact of OGT overexpression on hexokinase (HK), phosphoglycerate kinase (PGK), and pyruvate kinase (PK) activities. Although they have not evaluated their direct O-GlcNAcylation status, they showed that HK activity was increased while PGK and PK activities were decreased under those conditions. Champattanachai et al. (23) employed a proteomic strategy to compare the profile of *O*-GlcNAcylated proteins between benign and grade III breast tumors. They identified several proteins that were hyper-*O*-GlcNAcylated in cancer tissues; among them, four were glycolytic enzymes: enolase 2, triosephosphate isomerase, PK, and glyceraldehyde 3-phosphate dehydrogenase (GAPDH). They confirmed GAPDH and PKM2 O-GlcNAcylation by immunoprecipitation assays. Interestingly, GAPDH and PKM2 are also known for their non-glycolytic roles in cancer, which may also be regulated by *O*-GlcNAc.

### Glutamine

As well as glucose, glutamine (Gln) is an essential nutrient for many cancer cells. After entering the cell through specific transporters like SLC1A5, Gln can be consumed in the HBP pathway, but most of it is converted into glutamate (Glu) by glutaminases (Gls). Glu can then have three fates: it can be converted into α-ketoglutarate by glutamate dehydrogenase to produce Krebs cycle intermediates; it can be used to produce glutathione (GSH), together with glycine and cysteine; or can be converted to non-essential aminoacids by transaminases. Gls 1 and 2 expression is regulated by c-Myc and p53, respectively. c-Myc-overexpressing cells become Gln addicted since they have increased levels of GLS1 (41). PDAC cells utilize a different Gln pathway and have been shown to be extremely dependent on glucose and glutamine, but curiously not dependent on NADPH produced by the PPP. However, to deal with the damaging reactive oxygen species, they use a pathway depending on aspartate aminotransferase (Got) to convert aspartate into oxaloacetate, which is then converted into malate and finally into pyruvate by malic enzyme producing NADPH (42). Some cancer cells, however, are not dependent on the supply of Gln because they are able to synthesize their own from glucose. Glutamine synthetase (Glul) catalyzes the conversion of glutamate into glutamine and the dependence on this pathway seems to rely on the oncogene activated and the tissue of origin (41, 43). Glutamate dehydrogenase, both Got isoforms and Glul can be *O*-GlcNAcylated (39). These modifications may be exploited by cancer cells in favor of growth and metastasis.

### Krebs cycle and mitochondria

The connection between glycolysis and the Krebs cycle is performed by pyruvate dehydrogenase (PDH), which transforms pyruvate into acetyl CoA. PDH can be phosphorylated and inhibited by pyruvate dehydrogenase kinase (PDK), which is often up-regulated in cancer. Inhibition of PDH decreases mitochondrial function, contributing to the Warburg effect that is typical of cancer cells; PDK has been proposed as a therapeutic target in many cancers (44). O-GlcNAcylation of the alpha and beta subunits of PDH have also been demonstrated by Clark et al. (39) and as an important point of regulation, it would be interesting to understand its function. In 2003, Love et al. demonstrated that a smaller isoform of OGT was enriched in mitochondrial extracts. Further characterization showed a mitochondrial targeting sequence and a membrane domain in the N-terminus (45). However, mOGT overexpression induced apoptosis by an unclear mechanism and it was dependent on its catalytic activity (46). Clark et al. (39) demonstrated by 2D electrophoresis and mass spectrometry that many mitochondrial proteins in the rat forebrain could be *O*-GlcNAcylated. They identified the protein subunits alpha and beta of ATP synthase and two proteins from complex I: Ndufs 1 and Ndufs 2. In addition, the exposure of cardiac myocytes to high glucose medium increased O-GlcNAcylation of mitochondrial proteins, impaired mitochondrial function and led to decreased ATP pools. O-GlcNAcylation of the mitochondrial proteins COXI and NDUFA9 were identified by immunoprecipitation and both were more *O*-GlcNAcylated after exposure to high glucose (47). Tan et al. (48) also demonstrated that overexpression of OGT and OGA affected mitochondria morphology and mitochondrial localization of Krebs cycle and respiratory chain proteins leading to decreased respiration and glycolysis.

Importantly, mitochondria are dynamic organelles that can be remodeled by fusion or fission. These processes are controlled by mitofusin 1/2 (MFN1/2) and dynamin-related protein (Drp1), respectively. The role of these processes in cancer is not completely clear, but increased Drp1 expression has already been demonstrated in many tumors and may confer selective advantage to cancer cells (49–51). Drp1 can be *O*-GlcNAcylated in cardiac myocytes at threonine 585 and 586 after treatment with OGA inhibitors. These modifications decrease the phosphorylation of serine 637 and increases Drp1 translocation to the mitochondria and its activity. Increased Drp1 activity leads to mitochondria fragmentation and reduces membrane potential (52). Thus, increased mitochondria fragmentation and protein O-GlcNAcylation could be related in cancer.

## O-GlcNAcylation Regulation of Tumor Formation, Maintenance, and Dissemination

### *O*-GlcNAc in proliferation and survival of tumor cells

Tumor cell maintenance is essential for tumor development and *O*-GlcNAc has a substantial function in the proliferation and survival of tumor cells. Two different groups have demonstrated that O-GlcNAcylation could modulate the growth of prostate tumor cells. In the LNCaP cell line, siRNA-mediated OGT knockdown resulted in decreased proliferation compared with control-transfected cells (27). In the same direction, Lynch and colleagues showed, using a PC3-ML cell line, an 80% reduction in the anchorage-independent growth of OGT shRNA-expressing cells compared with control cells. The ability of OGT knockdown cells to grow in a three-dimensional culture was significantly impaired by 65% compared with control cells. However, non-transformed OGT knockdown cells RWPE-1 did not demonstrate significant changes of growth in a three-dimensional culture (26). An increase of *O*-GlcNAc by PUGNAc or siRNA against OGA in the thyroid tumor 8305C cell line also increased cell viability and proliferation (36). In addition, Ma and colleagues showed that pancreatic tumor cell line PDAC, silenced for OGT display, reduced cell proliferation and anchorage-independent growth. However, knockdown of OGT in non-transformed HPDE prostate cells did not affect their proliferation (35). These results indicate that O-GlcNAcylation is important for tumor cell growth and cancer cells appear to be specifically susceptible to loss of OGT, suggesting that this enzyme may be a potential future therapeutic target in chemotherapy.

Caldwell and co-workers demonstrated that mice injected with OGT knockdown breast tumor cells showed a fourfold reduction in tumor volume and mass when compared with injected control cells. At necropsy, 84% of mice injected with cells expressing scrambled shRNA developed visible tumors, against 40% of mice injected with cells containing OGT shRNAs. The authors pointed out that tumors that eventually grew from OGT knockdown cells restored OGT expression, suggesting a selective pressure against tumor cells that are deficient in OGT (22). Another study showed that pancreatic tumor cells with OGT knockdown injected into mice also resulted in greatly reduced tumor growth or even no tumor formation (35). These data indicate the importance of OGT in tumor cell growth *in vivo*.

In a model of 3D cell culture, Onodera et al. (53) demonstrated a direct relationship between glucose uptake and cell transformation. Knockdown of the glucose transporter Glut3 or lowering the levels of glucose concentration in the media led to decreased EGFR and MEK phosphorylation and β1 integrin expression as well as increased organization of the cells in acini. Using glycolysis inhibitor and PKM2 knockdown, they demonstrated PKM2 could interact with soluble adenylyl cyclase leading to activation of the EPAC/RAP1 pathway, responsible for regulating β1 integrin expression and cell polarization. However, this pathway did not interfere dramatically into EGFR and MEK activation. Further analysis showed increased glucose uptake led to the activation of these pathways through HBP pathway and that the phenotype could be reversed by addition of GFTP and OGT inhibitors.

Besides the link between O-GlcNAcylation and cell proliferation/growth, the relationship between *O*-GlcNAc and tumor cell survival were observed. The reduction of O-GlcNAcylation in prostate tumor cells lines decreased the expression of the anti-apoptotic protein Bcl-xL and induced the pro-apoptotic cleavage of caspases-9, and -3, suggesting that reducing *O*-GlcNAc levels leads to activation of the intrinsic apoptotic pathway (35). Thus, *O*-GlcNAc may have a role in protecting tumor cells from apoptosis. Interestingly, this protection appears to be specific to tumor cells, because reducing O-GlcNAcylation by OGT knockdown of non-transformed HPDE cells did not trigger apoptosis (35). In addition, Kanwal and colleagues observed the same protective role of *O*-GlcNAc modification in breast tumor cells. They demonstrated that increased O-GlcNAcylation in MCF-7 cells had inhibitory effects on Tamoxifen-induced cell death, while O-GlcNAcylation reduction sensitized tumor cells to Tamoxifen treatment (54).

Taken together, *O*-GlcNAc is involved in tumor formation, growth, and survival *in vivo*. Furthermore, high levels of *O*-GlcNAcylated proteins seem to promote tumor cell proliferation and, may contribute to the specific resistance of tumor cells to spontaneous apoptosis induced by chemotherapies. The acquisition of multidrug resistance (MDR) phenotype by tumors is a major cause of cancer treatment failure (55), and according to Kanwal’s work, *O*-GlcNAc levels correlate with the acquisition of this phenotype in breast tumor cells, but more studies using different cell types and tumors are needed to establish this relationship.

### *O*-GlcNAc in metastasis

A crucial event for tumor development and dissemination is the acquisition of invasiveness. Cells with this ability can invade and migrate, generating metastasis. A significant decrease of cell migration and invasion in three different breast cell lines with OGT knockdown was observed when compared with the negative control. Importantly, OGA inhibition markedly increased cell migration and invasion of breasts cell lines (21, 56), pointing to an important role for *O*-GlcNAc in migration. The treatment with OGT inhibitors of MCF-10A-ErbB2 and MDA-MB-231 breast tumor cells decreased the cell migration using Transwell assays (22). OGT knockdown in the PC3-ML prostate tumor cell line also reduced their ability to migrate when compared with control cells (26). Another study using prostate tumor cells LNCaP siRNA-mediated OGT knockdown also decreased tumor cell invasion when compared with control siRNA-transfected cells (27).

Gu et al. (21) showed that in 4T1 cells OGT knockdown do not display significant differences in primary tumor weights. However, a dramatic reduction of visible metastatic nodules on the lung surface was observed. Moreover, Lynch and co-workers injected into mice highly bone metastatic prostate tumor cells (PC3-ML), silenced or not for OGT; after 5 weeks, animals injected with OGT knockdown cells had fourfold less bone metastases to mandibles and hind limbs. Surprisingly, the histological analysis showed that the PC3-ML cells forming the metastatic focus display normal OGT levels (26). These results support the functional role for OGT in breast and prostate metastasis.

Proteins of the matrix metalloproteinase (MMP) family are involved in extracellular matrix degradation in physiological processes such as metastasis. However, the mechanism involving *O*-GlcNAc and the invasion of tumor cells is still poorly understood. Alterations in MMP proteins levels can be modulated by *O*-GlcNAc. Knockdown of OGT led to a significant decrease in the expression of MMP-2 at both the mRNA and protein level compared with controls in breast, liver, and prostate tumor cells (22, 26, 34) and OGA silencing resulted in the up-regulation of MMP-2 (34). The OGT knockdown of prostate tumor cells showed marked reductions of FoxM1 protein levels (26). It should be noted that FoxM1 is a transcription factor that regulates MMP-2 expression, suggesting that OGT regulates cancer cell invasion by modulating MMP-2 expression. In addition, MMP-9 also showed a 50% reduction in its expression in OGT knockdown cells (26), and MMP-1 and MMP-3 were increased when the O-GlcNAcylation level was enhanced and decreased when O-GlcNAcylation was reduced (34).

A recent work demonstrated the O-GlcNAcylation at serine 108 in cofilin, a major regulator of actin dynamics that drives cell motility. Breast tumor cell lines overexpressing OGT considerably increased cell mobility. When the OGT-overexpressing cells were silenced for cofilin, the OGT-enhanced cell mobility was almost entirely reversed. In addition, mutant cells expressing S108A cofilin, showed an apparent defect in invasion compared with the wild-type cells (56). These data suggest that cofilin modification by *O*-GlcNAc is involved in tumor cell mobility and invasion.

## Oncogenes, Tumor Suppressors, and Proteins Involved in Tumor Biology That are Regulated by *O*-GlcNAc

### c-Myc

c-Myc is a proto-oncogene that acts as transcription factor regulating cell cycle and replication. The c-Myc protein is expressed in normal cells at low levels, but overexpressed in transformed and proliferative cells (57). Deregulation of c-Myc gene expression is one of the most frequently encountered events in human cancer, and is implicated in over half of all malignancies (58). c-Myc is glycosylated at Thr58 (59), a known phosphorylation site that leads to c-Myc degradation (60). Proximal to this site, the phosphorylation of Ser62 stimulates its activity, promoting cell transformation (60). Although the exact influence of *O*-GlcNAc modification on c-Myc is not known, O-GlcNAcylation of Thr58 would reduce the phosphorylation of this site, stabilizing c-Myc. Recently, Itkonen and colleagues showed that OGT levels are correlated with c-Myc protein levels but not with c-Myc mRNA levels, indicating that the effect of OGT on c-Myc levels is in the stability of the protein (16). Thus, the effect of O-GlcNAcylation of c-Myc would improve its transformation capacity, favoring tumor growth, but studies are needed to observe the cell phenotype produced by c-Myc O-GlcNAcylation.

### p53

p53 is a tumor suppressor that is involved in DNA damage repair and the induction of apoptosis. The p53 mutation prevents the DNA repair and induction of apoptosis favoring tumor cell survival. The p53 gene is one of the most prevalent genes mutated in tumor cells, being mutated in more than 50% of human cancers. p53 is *O*-GlcNAcylated on Ser149 and this modification stabilizes p53 and decreases p53-MDM2 interactions, by decreasing the phosphorylation on Thr 155, which is a known site that induces ubiquitination and degradation (61). In addition, overexpression of OGA increases MDM2 phosphorylation at Ser 166 (62), which stimulates MDM2-p300 interactions and results in p53 degradation. Higher levels and stabilization of p53 by elevated *O*-GlcNAc would lead to a higher tumor suppression activity, disfavoring tumor progression; however, p53 in tumors is usually mutated and less functional, so stabilization of mutated p53 by glycosylation should not lead to a significant tumor suppression in most tumors. Indeed, some cancers can present p53 mutants that contain Pro or Phe instead of Ser at the position 149 (63, 64), disabling the presence of *O*-GlcNAc. So far, the impact of this change in p53 transcriptional activity is not known.

### Ras

Ras is a family of transmembrane GTPases involved in signal transduction that leads to cell proliferation. Mutations in RAS genes occur in 30% of all tumors (65) and lead to a constitutively activated Ras conformation. This oncogene is highly activated in human cancers appearing in 90% of pancreatic tumors (66) and 50% of colon tumors (67). K-Ras has a role in HBP as it controls GFAT levels; K-Ras depletion results in the inhibition of HBP and decreased protein O-GlcNAcylation (68). K-Ras appears to be an important regulator of glucose metabolism in tumor cells and has an involvement in O-GlcNAcylation cell status (69). Ying and colleagues (68) demonstrated a complete regression of fully established tumors 1 week after K-Ras extinction, underscoring the role of the HBP and hyper-O-GlcNAcylation in controlling tumor maintenance.

### AMPK

AMPK is an important energy sensor enzyme responsible for regulating cellular metabolism in response to substrate availability. It is activated by AMP and ADP, while it is inhibited by ATP. Its activation enhances catabolic pathways and decreases many anabolic pathways important for cancer cell growth, making of it an excellent therapeutic target. It is a heterotrimeric enzyme composed of a catalytic subunit α and regulatory subunits β and γ. There are several isoforms for each subunit, which can interact to form 12 different complexes (70). Since *O*-GlcNAc signaling can also act as an energy sensing pathway, a significant crosstalk between them is expected although it has not been deeply exploited yet.

Neuronal glucose deprivation leads to up-regulation of OGT expression and increased O-GlcNAcylation of proteins in a AMPK pathway dependent manner (71). In myotubes, Bullen et al. (72) demonstrated AMPK phosphorylates T444 of OGT *in vitro*, which correlates with OGT nuclear accumulation. In addition, OGT can also *O*-GlcNAcylate AMPK subunits α and γ and inhibition of *O*-GlcNAc cycling by TMG treatment mitigate AMPK activation. Finally, they have also demonstrated, in proliferating cells, that AMPK activation affects OGT substrate selectivity (72).

### NF-κB

NF-κB is a transcription factor involved in cell proliferation, survival, and inflammation. This pathway can be activated in normal cells in certain occasions, but abnormal constitutive NF-κB activation has been linked to oncogenic growth/survival of many cancer types (73). Five NF-κB family members have been identified in humans but the most prevalent and best studied is a dimer composed of p65 (RelA) and p50 subunits. In unstimulated cells, this dimer is localized in the cytoplasm where it binds its inhibitor (IκB). Treatment with TNFα or other activating agents stimulates IκB kinase (IKK), which phosphorylates IκB inducing its degradation via an ubiquitin-dependent proteolysis. Free NF-κB translocates to the nucleus and activates the expression of target genes (74).

It is well established that NF-κB is modulated by the PTMs by phosphorylation, ubiquitination, and acetylation. However, growing evidences demonstrates that O-GlcNAcylation of NF-κB is also related to its activation. NF-κB subunit p65 (RelA) is *O*-GlcNAcylated on the Thr322 and Thr352 residues (35, 75), but only modification on Thr352 is important for NF-κB transcriptional activity (75). Further, site-directed mutagenesis of the threonines 322 and 352 of p65 led to decreased colony formation (35). Another study shows the presence of *O*-GlcNAc on Thr305 of NF-κB. The p65 (T305A) mutant is not acetylated by p300, linking the *O*-GlcNAc and acetylation events. Reconstitution of RelA null cells with the RelA (T305A) mutant are deficient for NF-κB transcription, and are more sensitive to TNF-induced apoptosis, indicating the importance of this residue for NF-κB-dependent gene expression and cell survival (76). In addition, the increase in glucose uptake by tumor cells results in O-GlcNAcylation of the IKKβ subunit at Ser733 causing IkB degradation, and maintaining NF-κB in the constitutively activated form in tumor cells (77). Hyper-O-GlcNAcylation, through the pharmacological inhibition of OGA, increases IKKα O-GlcNAcylation, accompanied by the activation of NF-κB signaling (35). These data propose a new regulation in tumor cells that promotes tumor growth and survival by the maintenance of the over activated NF-κB pathway in these cells.

## Conclusion and Future Directions

Taken together, *O*-GlcNAc appears to be important in tumor biology, and altered O-GlcNAcylation is an event found in many types of cancer. This aberrant O-GlcNAcylation correlates with augmented tumor cell proliferation and survival, increased tumor formation, invasion, metastasis, and resistance to therapy. These events point to several interesting conclusions: (1) *the use of O-GlcNAcylation as diagnosis factor*: once transformed cells exhibit higher or lower levels of this PTM, the accurate determination of this level change may be a biomarker of cellular malignancy in some tumor types. However, further studies should be conducted to validate it. Importantly, the levels of protein glycosylation are not determinant to characterize a transformed cell, but its variation compared to normal tissue. Remembering that the amount of *O*-GlcNAc levels in tumor cells varies from tissue to tissue, establishing specific parameters would therefore be important for each tumor type. (2) *The use of O-GlcNAcylation as prognostic factor*: the literature already describes that O-GlcNAcylation could be used as a prognostic factor for some types of tumor as for, e.g., prostate and leukemia. Once parameters for each tissue are established it will be possible to speculate about the tumor grade, tumor proliferation, survival and response to treatment of these patients. In addition, the search for aberrant O-GlcNAcylation of specific key proteins may help the discovery of new cancer markers or prognostic factors. (3) *O-GlcNAcylation as therapeutic target in cancer*: many metabolic pathways intersect on UDP-GlcNAc synthesis pathway, HBP, making it an important metabolic sensor. Considering metabolic reprograming as a hallmark of cancer and the evidence showing that normal cells are less influenced by interventions in the levels of O-GlcNAcylation, is possible to suggest that therapies targeting the *O*-GlcNAc enzymes, OGT and OGA, or the *O*-GlcNAcylated form of specific proteins may have therapeutic potential. Importantly, the lower sensitivity to *O*-GlcNAc manipulation in normal cells may lead to fewer side effects than many chemotherapy drugs used in the clinic today, improving the quality of life of patients. For the development of *in vivo* studies, it is important to remember that OGT and OGA knockouts are not viable. One possible solution for this impasse would be the development of more efficient and specific inhibitors of OGT and OGA. The choice of an appropriate concentration could decrease or impair cancer cell proliferation minimizing adverse effects to normal cells. In addition, studies aimed at obtaining specific inhibitors of OGT that act on key target proteins are important for further progress in this area.

However, for the application of any of the alternatives mentioned, larger studies are needed to define the profile of *O*-GlcNAc modifications for each type of tissue and its relationship to diagnosis and prognosis, in addition to studies seeking novel proteins important in tumor signaling that are modified by *O*-GlcNAc. It is not known whether increased O-GlcNAcylation is the cause or consequence of cancer, and it is still too early to indicate the exact role of O-GlcNAcylation in the pathology of different cancer types. However, collectively, these studies indicate that *O*-GlcNAc has potential in diagnosis, prognosis evaluation and as a therapeutic target in cancer.

## Conflict of Interest Statement

The authors declare that the research was conducted in the absence of any commercial or financial relationships that could be construed as a potential conflict of interest.
